# Drug screening on Hutchinson Gilford progeria pluripotent stem cells reveals aminopyrimidines as new modulators of farnesylation

**DOI:** 10.1038/cddis.2015.374

**Published:** 2016-02-18

**Authors:** S Blondel, A-L Egesipe, P Picardi, A-L Jaskowiak, M Notarnicola, J Ragot, J Tournois, A Le Corf, B Brinon, P Poydenot, P Georges, C Navarro, P R pitrez, L Ferreira, G Bollot, C Bauvais, D Laustriat, A Mejat, A De Sandre-Giovannoli, N Levy, M Bifulco, M Peschanski, X Nissan

**Affiliations:** 1 INSERM U861, I-STEM, AFM, Institute for Stem Cell Therapy and Exploration of Monogenic Diseases, 5 rue Henri Desbruères, Evry Cedex 91030, France; 2 UEVE, I-STEM, AFM, Institute for Stem Cell Therapy and Exploration of Monogenic Diseases, 5 rue Henri Desbruères, Evry Cedex 91030, France; 3 CECS, I-STEM, AFM, Institute for Stem Cell Therapy and Exploration of Monogenic Diseases, 5 rue Henri Desbruères, Evry Cedex 91030, France; 4 Department of Medicine and Surgery, University of Salerno, Via Allende, Baronissi Salerno 84081, Italy; 5 Laboratory of Nutritional Biochemistry, National Institute for Digestive Diseases “S. de Bellis”, Castellana Grotte, Bari 70013, Italy; 6 Aix Marseille Université, UMR S 910: Génétique Médicale et Génomique Fonctionnelle, Faculté de Médecine Timone, Marseille, France; 7 INSERM, UMR S 910: Génétique Médicale et Génomique Fonctionnelle, Faculté de Médecine, Marseille, France; 8 CNC-Center for Neurosciences and Cell Biology, University of Coimbra, Largo Marques de Pombal, Coimbra 3004-517, Portugal; 9 SYNSIGHT, a/s IncubAlliance 86 rue de Paris Orsay 91400, France; 10 Ecole Normale Supérieure de Lyon, Laboratoire de Biologie Moléculaire de la Cellule, UMR 5239 CNRS/ENS Lyon/UCBL, 46 Allée d'Italie, Lyon, France

## Abstract

Hutchinson-Gilford progeria syndrome (HGPS) is a rare genetic disorder characterized by a dramatic appearance of premature aging. HGPS is due to a single-base substitution in exon 11 of the *LMNA* gene (c.1824C>T) leading to the production of a toxic form of the prelamin A protein called progerin. Because farnesylation process had been shown to control progerin toxicity, in this study we have developed a screening method permitting to identify new pharmacological inhibitors of farnesylation. For this, we have used the unique potential of pluripotent stem cells to have access to an unlimited and relevant biological resource and test 21 608 small molecules. This study identified several compounds, called monoaminopyrimidines, which target two key enzymes of the farnesylation process, farnesyl pyrophosphate synthase and farnesyl transferase, and rescue *in vitro* phenotypes associated with HGPS. Our results opens up new therapeutic possibilities for the treatment of HGPS by identifying a new family of protein farnesylation inhibitors, and which may also be applicable to cancers and diseases associated with mutations that involve farnesylated proteins.

Progeria, also known as Hutchinson-Gilford progeria syndrome (HGPS), is a rare, fatal genetic disease characterized by an appearance of accelerated aging in children (OMIM #176670).^1^ This syndrome is due to a single base substitution in exon 11 of the *LMNA* gene^2, 3^ (c.1824C>T, NCBI Reference Sequence: NM_170707.3), which activates a cryptic splicing donor site, leading to the production of a truncated form of the prelamin A protein called progerin.^4^ Because the deleted sequence is required for its posttranslational maturation, this mutant protein accumulates at the nuclear membrane, disrupting the shape of the nucleus and producing a set of well-characterized cellular dysfunctions, including premature senescence and defects in DNA repair, cell proliferation and differentiation.

Since the discovery of the molecular mechanisms underlying HGPS, three different drugs have been repurposed for their ability to target the prenylation process, namely the HMG-CoA reductase (HMGCR) inhibitor pravastatin combined with the aminobisphosphonate zoledronate, which inhibits farnesyl pyrophosphate synthase (FPPS), and the farnesyl transferase inhibitor (FTI) lonafarnib.^5, 6, 7^ Over the past 10 years, several *in vitro* studies have demonstrated the potential of these pharmacological approaches, showing that inhibition of prelamin A prenylation correlated with the improvement in nuclear shape and other HGPS-related cellular defects.^7, 8, 9, 10^ *In vivo* testing of several prenylation inhibitors in various animal models of HGPS^5, 6, 11, 12^ subsequently confirmed the therapeutic potential of this strategy, prompting three clinical trials. Data from one of these trials have been reported and indicate some partial improvements in the patients' clinical phenotypes, highlighting in addition the need for new potential drugs.^13^ However, until now, mainly because of the premature senescence of primary HGPS cells, the lack of appropriate *in vitro* cellular models has precluded high-throughput screening (HTS) of chemical compounds.

The pluripotency and self-renewal properties of induced pluripotent stem (iPS) cells offer a unique way to produce an unlimited and homogeneous biological resource for testing chemical compounds *in vitro*, in a HTS setting.^15, 16^ Since 2011, several groups, including ours, have demonstrated the capacity of iPS cell lines to recapitulate some aspects of HGPS after differentiation into vascular smooth muscle cells (VSMCs) and mesenchymal stem cells (MSCs).^17, 18, 19^ In 2014, our group showed that these cells could be used to evaluate *in vitro* the functional effects of the drugs that are currently used in HGPS patients on typical cellular and molecular defects, such as nuclear shape architecture, progerin expression and premature differentiation along the osteoblastic lineage.^20^ More recently, Soria-Valles *et al.*^21^ reported that the generation of iPS cells from cells of patients with HGPS or Nestor-Guillermo progeria syndrome is impaired due to NF-*κ*B hyperactivation, which elicits the reprogramming repressor DOT1L. Finally, this groundbreaking study described DOT1L inhibition as a novel therapeutic strategy for progeroid syndromes, showing that it extends lifespan and counteracts the accelerated ageing phenotype of progeroid mice.^21^

In this study, we used iPS cell lines derived from HGPS patients to test a large number of chemical compounds by HTS. This approach identified aminopyrimidines (AP), and in particular mono-aminopyrimidines (Mono-AP), as a chemical family hitherto unknown to be capable of restoring several HGPS-related pathological cellular phenotypes.

## Results

### Identification of inhibitors of prelamin A farnesylation by HTS

**Figure 1:**
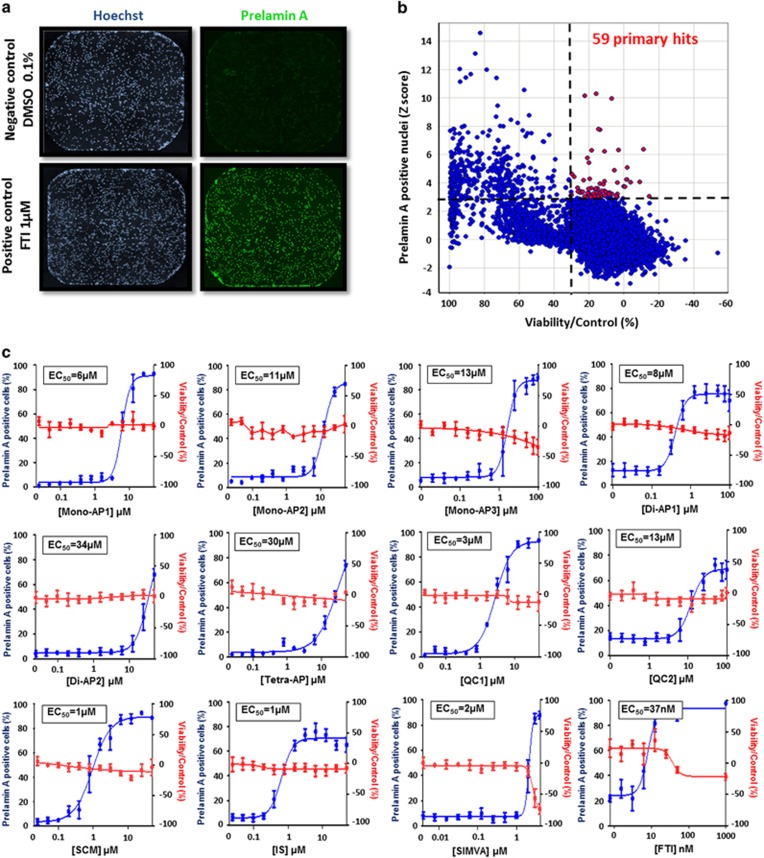
High-throughput screening of 21 608 small molecules on prelamin A maturation. (**a**) Prelamin A and Hoechst immunostaining in HGPS MSCs treated with FTIs. (**b**) Primary screen cell-based assay for prelamin A localization. Dot plot representation of the effects of the 21 608 compounds on prelamin A maturation process and cell viability. (**c**) Dose-response experiments of the 11 prelamin A modulators identified. Each chart represents cell viability (in red) and percentage of prelamin A-positive nuclei (in blue). Each point represents the mean±S.D. of the percentage of eight replicates

This study was performed on MSCs derived from HGPS iPS cells (HGPS MSCs), differentiated as previously described^20^ and used after amplification through 10 passages (Supplementary Figures 1a and d). The drug screening strategy was based on the detection of the subcellular localization of prelamin A, with the assumption that its accumulation within the nuclear compartment was directly correlated to the inhibition of its farnesylation. A high-throughput immunostaining assay was developed to robustly quantify prelamin A content and its localization in 384-well plates (Figure 1a, Supplementary Figure 1e). DMSO 0.1% and a FTI (tipifarnib 1 *μ*M) were used as negative and positive controls, respectively. The drug screening assay was optimized in order to obtain homogeneous and reproducible prelamin A staining in HGPS MSCs treated with FTIs in 384-well plates (Supplementary Figures 1f and g). Quality control of the assay was assessed by the calculation of a Z′ factor superior to 0.8, indicating a good separation between the negative and positive controls (Supplementary Figure 1g). 21 608 small molecules from 4 different compound libraries (Supplementary Figure 2a) were then tested, following the various steps of the screening procedure (Supplementary Figure 2b). Prelamin A localization was quantified using an automated imaging platform that measures the proportion of HGPS MSCs exhibiting prelamin A staining in the nuclear matrix compartment 48 h after pharmacological treatment.

**Figure 2:**
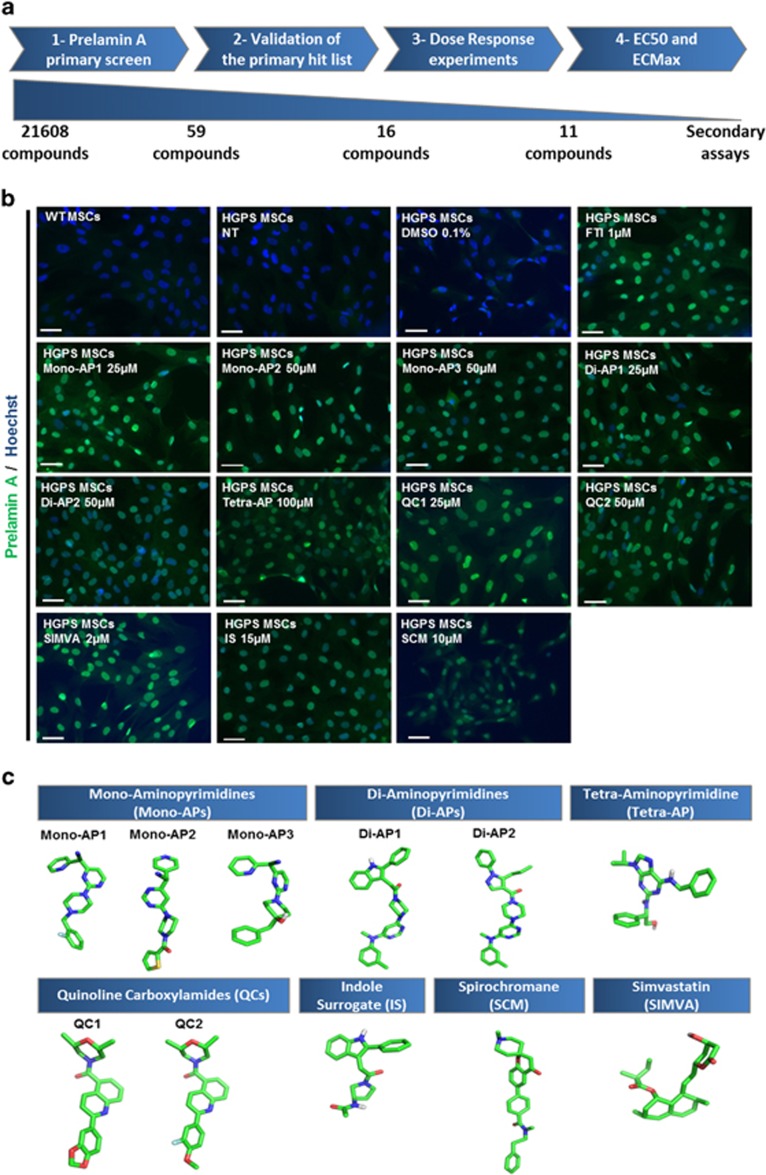
Results of the screening of prelamin A modulators on HGPS MSCs. (**a**) Schematic representation of the four-step analysis leading to the identification of the hits list. (**b**) Prelamin A immunostaining of HGPS MSCs following 48 h of treatments with each of the 11 validated compounds. Scale bar represents 50 *μ*m. (**c**) 3D chemical structures of considered molecules for secondary investigations. For clarity, only polar hydrogen atoms are represented

Compounds were considered as potential candidates when their effect was superior to 3 S.D. from the mean of all the tested compounds without affecting cell viability by more than 30% (Figure 1b). This led to an initial list of 59 hits (Supplementary Table 1). Retest experiments excluded 43 of these candidates because they were either toxic or deemed false positives (Supplementary Table 1). The efficacy and toxicity of these 16 validated compounds were then evaluated at progressively higher doses, revealing that 11 exhibited greater than 50% efficacy without cell toxicity (Figure 1c). The EC_50_ of these compounds ranged from 1 to 34 *μ*M, with a plateau at the highest nontoxic doses at which over 80% of cells were positive for nuclear prelamin A localization (Figure 1c). The optimal concentration was determined for each compound as the highest effective concentration on prelamin A nuclear localization without toxicity, and was used subsequently for secondary and functional assays (Figures 2a and b and Supplementary Figure 2c).

**Figure 3:**
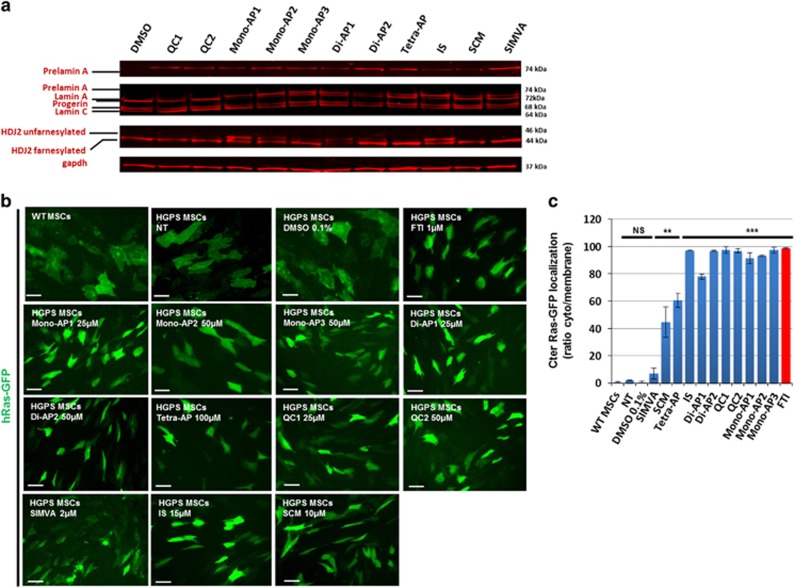
Effect of the 11 validated compounds on farnesylation process. (**a**) Western blot analysis of Lamin A, lamin C, progerin, prelamin A and HDJ2 expression in HGPS MSCs following 48 h of treatment with each of the 11 validated compounds. (**b**) GFP localization of HGPS MSCs overexpressing hRas-GFP following 48 h of treatment with each of the 11 validated compounds. Scale bar represents 50 *μ*m. (**c**) Quantification of the percentage of HGPS MSCs presenting a cytoplasmic (unfarnesylated) localization of GFP following 48 h of treatments with each of the 11 prelamin A modulators. Each chart represents the mean±S.D. of three independent experiments

Chemical structure analysis of the 11 hits (Figure 2c) showed that 6 of them contained an AP group and also one, two or four nitrogen atom bindings, respectively, named Mono-APs, Di-APs and Tetra-APs. The others were two quinoline carboxamides (QCs), one statin (SIMVA), one indole surrogate (IS) and one spirochromane (SCM). Measures of cell toxicity demonstrated that none of these compounds were toxic at the effective doses for prelamin A farnesylation, except SIMVA. Western blot analyses confirmed that prelamin A was increased in cells treated with each of the 11 compounds (Figure 3a), whereas quantitative PCR confirmed the posttranslational effect of these drugs showing that, apart from two (SCM and Di-AP1), neither lamin A nor progerin mRNAs were increased after the various treatments (Supplementary Figures 3a and b). Immunostaining confirmed this result, showing that none of these drugs affected progerin expression at the protein level (Supplementary Figure 3c). Finally, the farnesylation status of two other prenylated proteins, HDJ2 and H-Ras, was determined, showing an increase of the unfarnesylated form of HDJ2 after treatment with Mono-AP1, Mono-AP2, Mono-AP3, Di-AP1, Di-AP2, QC1, QC2 or IS (Figure 3a) and inhibition of H-Ras farnesylation in the presence of all compounds except SIMVA (Figures 3b and c).

### Functional effects of the 11 hits on HGPS-related defects

**Figure 4:**
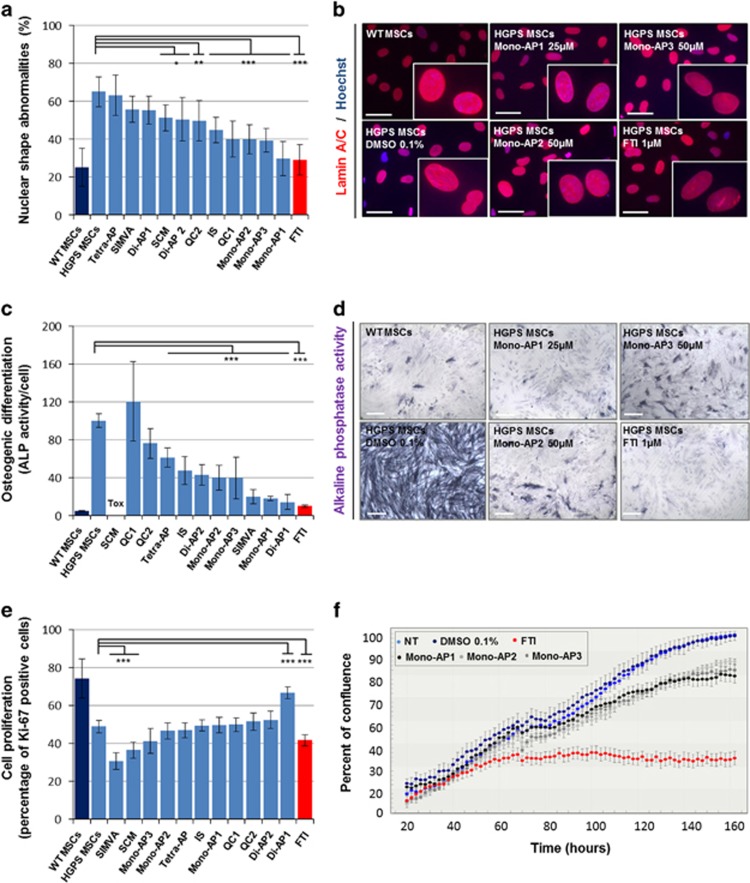
Pharmacological evaluation of the 11 prelamin A modulators on HGPS defects. (**a**) Measure of nuclear shape abnormalities (lamin A/C immunostaining) in HGPS MSCs following 48 h of treatment with each of the 11 prelamin A modulators. Each chart represents the mean±S.D. of 8 independent experiments. (**b**) Lamin A/C immunostaining of WT and HGPS MSCs following 48 h of treatments with each of the three Mono-APs (Mono-AP1, Mono-AP2 and Mono-AP3). Scale bar represents 50 *μ*m. (**c**) Measure of osteogenic differentiation (Alkaline phosphatase activity) in HGPS MSCs following 7 days of differentiation in presence of each of the 11 prelamin A modulators. Each chart represents the mean±S.D. of eight independent experiments. Data are normalized on cell number. (**d**) Alkaline phosphatase activity of WT and HGPS MSCs following 7 days of differentiation in presence of each of the three Mono-APs (Mono-AP1, Mono-AP2 and Mono-AP3). Scale bar represents 50 *μ*m. (**e**) Measure of cell proliferation (Ki-67 immunostaining) in HGPS MSCs following 48 h of treatments with each of the 11 prelamin A modulators. Each chart represents the mean±S.D. of eight independent experiments. (**f**) Measure of percent of confluence in long-term culture in presence of Mono-APs (Mono-AP1, Mono-AP2 and Mono-AP3)

Because the inhibition of protein farnesylation has been reported to improve nuclear shape abnormalities in HGPS cells, the 11 hits were tested for their capacity to rescue this pathological phenotype. HGPS MSCs were treated for 48 h with these compounds and stained for lamin A/C. Quantification of abnormal nuclei revealed that Mono-AP1, Mono-AP2 and Mono-AP3 were the three most effective compounds for restoring this pathological structural defect. Di-AP2, QCs and IS also significantly restored this defect, whereas Di-AP1, Tetra-AP and SIMVA were not effective (Figures 4a and b, Supplementary Figure 4a). The effect of Mono-APs was confirmed in wild-type (WT) and HGPS primary fibroblasts, in which similar improvements were observed (Supplementary Figures 4b and 4c).

Because HGPS MSCs exhibit premature differentiation into the osteoblastic lineage,^20, 22^ we then evaluated the effect of the 11 hits on this functional impairment by measuring alkaline phosphatase activity after 7 days of osteogenic differentiation. All the compounds except QC1 and SCM strongly rescued this phenotype, and the efficacy of Mono-AP1, Di-AP1 and SIMVA was not significantly different from that observed with FTI treatment (Figures 4c and d).

Potential adverse effects of the 11 compounds were assessed by measuring the proliferation of treated HGPS MSCs, revealing decreased proliferation with SIMVA and SCM treatment, but not with the other compounds (Figure 4e). Interestingly, Di-AP1 actually increased the number of Ki-67-positive cells to 65%. These findings were confirmed in long-term live imaging cultures, which showed that Mono-AP1, Mono-AP2 and Mono-AP3 were less cytostatic than the FTI tipifarnib (Figure 4f).

### Mode of action of Mono-APs

Because Mono-APs were the most effective compounds for rescuing the two functional defects tested without inducing adverse effects, the next part of this study focused on identifying their mechanism of action.

**Figure 5:**
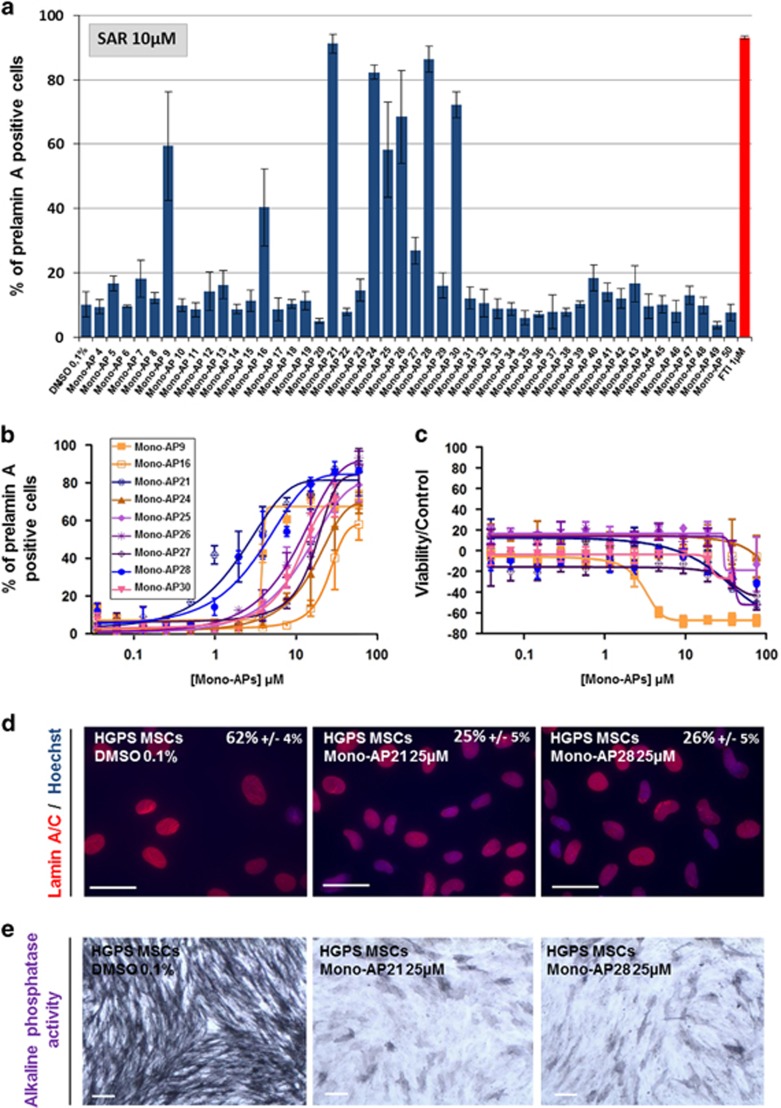
Structure–activity relationship of Mono-APs. (**a**) Automated quantification of prelamin A-stained nuclei in HGPS MSCs following 48 h of treatment with 47 compounds containing a Mono-APs domain at 10 *μ*M. Data are compared with FTI 1 *μ*M (in red). Each value represents the mean±S.D. of the percentage of four replicates. (**b**) Dose-response analysis of the nine hits identified as positives (Mono-AP21, Mono-AP28, Mono-AP26, Mono-AP9, Mono-AP30, Mono-AP25, Mono-AP27, Mono-AP16, Mono-AP24) on prelamin A maturation process in HGPS MSCs. Each point represents the mean±S.D. of the percentage of eight replicates. (**c**) Cellular viability of HGPS MSCs after the treatment with the nine hits identified as positives (Mono-AP21, Mono-AP28, Mono-AP26, Mono-AP9, Mono-AP30, Mono-AP25, Mono-AP27, Mono-AP16, Mono-AP24). Each point represents the mean±S.D. of the percentage of eight replicates. (**d**) Measure of nuclear shape abnormalities (lamin A/C immunostaining) in HGPS MSCs following 48 h of treatment with Mono-AP21 25 *μ*M and Mono-AP28 25 *μ*M. Values represent the mean±S.D. of three independent experiments. (**e**) Alkaline phosphatase activity of HGPS MSCs following 7 days of differentiation in the presence of Mono-AP21 25 *μ*M and Mono-AP28 25 *μ*M

To achieve this, a library of 47 chemical analogs was derived from Mono-AP1 ({2-[4-(2-Fluoro-benzyl)-piperazin-1-yl]-pyrimidin-4-yl}-pyridin-4-yl-acetonitrile; Supplementary Figure 5a and Supplementary Table 2), and each evaluated at a 10 *μ*M concentration for their ability to inhibit prelamin A maturation. These experiments revealed that the effect of nine compounds on prelamin A localization was greater than 20% (Figure 5a). Dose-response experiments with these nine second-generation Mono-APs showed that only two of them, Mono-AP21 and Mono-AP28 were more effective than Mono-AP1, with EC_50_s of 1.5 and 2 *μ*M, respectively (Figures 5b and c). After treatment with Mono-AP21 and Mono-AP28, a similar capacity for rescuing nuclear shape defects and premature osteogenesis in HGPS MSCs was observed (Figures 5d and e).

Structural analysis of the entire set of Mono-AP1 analogs showed that those lacking a piperazine nucleus, as in Mono-AP5, 17, 18 and 19, were less effective than those that contained this moiety (Figure 5a, Supplementary Table 2). This suggests that the nitrogen in position 4 of the piperazine nucleus (N4) is necessary. This nitrogen atom has to have sufficient basicity for significant efficacy to be retained, as activity was lost when it was acetylated, as illustrated by Mono-AP 41, 44, 45 and 46 (Figure 5a, Supplementary Table 2). Efficacy fell when the benzyl group attached to the piperazine nucleus was substituted with a phenyl group, as in Mono-AP11, or with a functionalized chain such as –CH_2_CO_2_Et, as in Mono-AP25 (Supplementary Figure 5b). Finally, the simple deletion of the nitrile residue of Mono-AP1 abolished its biological activity (Mono-AP50), suggesting a major role for this nitrile residue in the inhibition of prelamin A farnesylation (Supplementary Figure 5c).

**Figure 6:**
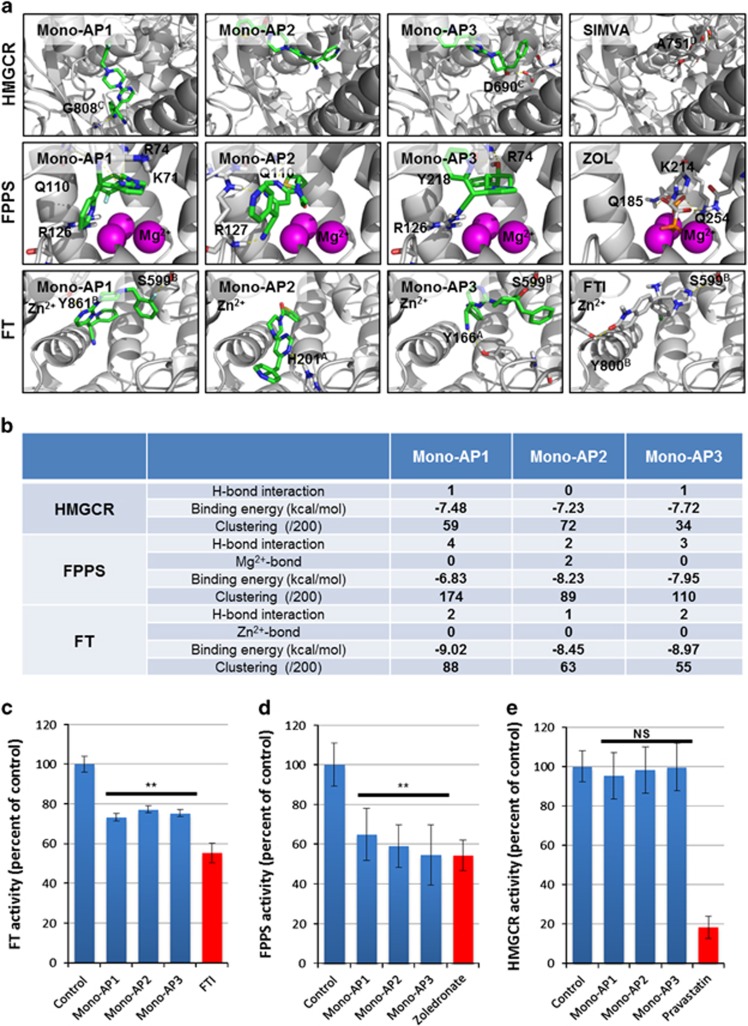
Molecular docking of Mono-APs on HMG-CoA reductase (HMGCR), farnesyl pyrophosphate synthase (FPPS) and farnesyl transferase (FT). (**a**) Molecular docking representation of complexes between HMGCR (up), FPPS (middle) and FT (down) enzymes and compounds belonging to the Mono-AP family (Mono-AP1, Mono-AP2 and Mono-AP3) and their respective positive control molecules simvastatin (SIMVA), zoledronate (ZOL) and the tipifarnib (FTI). Enzymes are represented in white ribbons and interacting amino acids are in white thin sticks. Mono-APs are illustrated in green sticks and positive control molecules are in gray sticks. Cations Mg^2+^ and Zn^2+^ are represented in sphere and colored in magenta and black, respectively. (**b**)Table of molecular docking results with Mono-APs molecules showing the number of hydrogen bonds, interactions with cations, calculated binding energies (kcal/mol), and clustering. (**c**) Measure of FT activity in presence of Mono-AP1 25 *μ*M, Mono-AP2 50 *μ*M and Mono-AP3 50 *μ*M. Tipifarnib 1 *μ*M (FTI) was used as positive control. Results are presented in percent of control. Each point represents the mean±S.D. of the percentage of 8 replicates. (**d**) Measure of FPPS activity in presence of Mono-AP1 25 *μ*M, Mono-AP2 50 *μ*M and Mono-AP3 50 *μ*M. Zoledronate 1 *μ*M was used as positive control. Results are presented in percent of control. Each point represents the mean±S.D. of the percentage of 8 replicates. (**e**) Measure of HMGCR activity in presence of Mono-AP1 25 *μ*M, Mono-AP2 50 *μ*M and Mono-AP3 50 *μ*M. Pravastatin 1 *μ*M was used as positive control. Results are presented in percent of control. Each point represents the mean±S.D. of the percentage of eight replicates

Finally, in order to highlight potential avenues for optimizing these compounds and to validate their specific effect on prelamin A farnesylation, we virtually assessed direct interactions of Mono-AP1, Mono-AP2 and Mono-AP3 with three key enzymes controlling protein prenylation: HMGCR, FPPS and FT (Figures 6a and b). The calculated binding energies of Mono-APs with the three enzymes indicated potential interaction with the active sites of FPPS and FT, but not HMGCR (Figure 6b). Docking of Mono-AP1 and Mono-AP3 on FPPS revealed the major role of the phenyl domain of these compounds for cation–pi interaction with the magnesium cation of FPPS (Figure 6a, Supplementary Videos 1–4). Appropriate substitution, such as with an electron donating group (Me, OMe or NH_2_), may strengthen the binding of the phenyl domain of Mono-AP3 to the magnesium cation (Figure 6a). This study also revealed that Mono-AP1 showed a potential higher affinity for FT than Mono-AP2 and Mono-AP3, because of the presence of hydrogen bonds with amino acids Y861B and S599B (Figure 6a). Mono-APs interactions with FPPS were confirmed using surface plasmon resonance (SPR; Supplementary Figure 6a). FPPS was immobilized on a CM5 chip and its interaction with Mono-APs was measured using a Biacore platform. These experiments confirmed the direct interactions of Mono-AP1 and Mono-AP2 with FPPS, and equilibrium dissociation (Kd) and association (Ka) constants were calculated for both interactions (Supplementary Figure 6b). It was not possible to evaluate the interaction of Mono-AP3 with FPPS because it precipitated in the HBSEP-5% DMSO buffer used to load the chip. The interactions of Mono-APs with FT could not be determined either, due to similar issues (data not shown).

Our molecular docking and SPR findings were confirmed by measuring the biochemical activities of these three enzymes in the presence of Mono-APs. The Mono-APs directly inhibited both FPPS and FT but not HMGCR, with a 20% to 30% decrease in FT activities and a 40% to 50% decrease for FPPS activities (Figures 6c and e). Finally, dose-response experiments showed that the IC_50_ of Mono-AP1, 2 and 3 on FT and FPPS activities ranged from 0.35 to 9.32 *μ*M (Supplementary Figure 6c and Supplementary Figure 6d).

## Discussion

The main finding of this study is the discovery of a new family of farnesylation inhibitors capable of rescuing pathological phenotypes associated with HGPS (Supplementary Figure 7). In addition to their potential use for the treatment of HGPS, these compounds could represent a new class of molecules for disorders already treated with farnesylation inhibitors. Moreover, our study also highlights the unique potential of iPS cell derivatives for drug discovery.

The persistence of an abnormal farnesylated motif at the C-terminal end of misprocessed prelamin A is the main molecular mechanism underlying progerin toxicity in HGPS.^7, 23^ This discovery has allowed to demonstrate the potential therapeutic value of several known inhibitors of prenylation, leading to independent preclinical and clinical studies with FTIs and a combination of a bisphosphonate and a statin.^5, 11, 13^ The results of the clinical trials of FTI-based therapy have revealed some limited improvements in patients but underscored the need for new, more effective and less toxic compounds.^13^ The present study was based on the assumption that such compounds could be identified by measuring their effects on prelamin A prenylation in HGPS cells. We, therefore, monitored prelamin A accumulation and localization, and confirmed the therapeutic potential of a number of compounds for HGPS, subsequently measuring their beneficial effect on two pathological phenotypes, that is, nuclear shape disruption and premature differentiation. Although nuclear shape disruption is not specifically observed in HGPS, this phenotype remains the gold standard when measuring progerin-induced structural defects, and is improved by FTIs.^7^ In 2014, our group revealed premature osteoblastic differentiation of HGPS MSCs,^20^ in line with previous reports describing that overexpression of progerin leads to increased osteogenic differentiation of MSCs.^22^ Similarly, Villa-Bellosta *et al.* recently described increased alkaline phosphatase expression and activity in progerin-expressing VSMCs and demonstrated that the vascular calcification observed in this syndrome is due to defective extracellular pyrophosphate metabolism.^24^ Together, these studies, as well as ours, suggest that calcification and alkaline phosphatase activity are relevant readouts for evaluating the potential value of drugs in HGPS.

Interestingly, 3 of the 11 hits obtained in our screen of 21 608 small molecules – one statin and two quinolines – had already been identified in other studies as prelamin A farnesylation modulators.^5, 7^ In fact, QCs were originally described as inhibitors of Ras farnesylation, and their therapeutic use as antiproliferative agents in cancer was therefore suggested.^25^ QCs have also been evaluated in patients with malaria for their ability to inhibit FT in plasmodium falciparum,^26, 27, 28, 29^ then later, based on their ability to interfere with farnesylation, were tested for their ability to improve nuclear blebbing in fibroblasts derived from HGPS patients.^8^ It has also been claimed that statins, which are widely prescribed in humans as HMGCR inhibitors to reduce cholesterol levels and prevent cardiovascular disorders,^30^ may have potential as HGPS treatments.^5^ In this previously reported HGPS studies, pravastatin was used in combination with zoledronate to inhibit protein prenylation and improve pathological phenotypes associated with this syndrome both *in vitro* and *in vivo*. In our study, we show that another statin, simvastatin, inhibits prelamin A farnesylation in the absence of zoledronate, but failed to demonstrate any functional benefit on the pathological defects tested, suggesting that its combination with zoledronate may be required.

The most striking result of our HTS was the fact that most of the compounds identified (6 out of 11) shared a common chemical structure, an AP group. Subsequent analysis of 47 related chemicals supported this finding, adding 9 other compounds to the initial list. Due to the experimental design used, compounds that caused accumulation of prelamin A in HGPS MSCs could either inhibit its farnesylation or promote cleavage of the farnesylated cysteine residue by ZMPSTE24. Although different targets and modes of action for APs have been described in the literature, it is interesting to note that other recent studies have highlighted the therapeutic potential of this family of compounds. Over the past 10 years, APs have been described as highly potent inhibitors of B-Raf,^31^ JAK,^32^ CDK1/2 inhibitors^33^ or EGFR and ErbB-2.^34^ An AP, nilotinib, was also reported to inhibit Bcr-Abl, the target of first-line therapy for most patients with chronic myelogenous leukemia.^35^ Docking studies have suggested that pyrimidine analogs may bind geranyl transferase, suggesting the involvement of pyrimidines in the regulation of protein prenylation.^36^ The addition of the pyrimidine group thienopyrimidine to bisphosphonates has also been shown to increase their binding to the allylic subpocket of FPPS.^37^ In accordance with this literature, our results show that APs can inhibit protein prenylation and are able to regulate this process in the absence of bisphosphonates, reducing FT and FPPS activities by 20% and 40%, respectively. This was supported by our docking results, which showed that Mono-APs display strong affinity for the active sites of FPPS and FT.

Since the discovery of the protein prenylation mechanism in fungi in 1978,^38^ more than 100 proteins have been confirmed experimentally to undergo prenylation, including the small GTPases Rho, Ras, Rac and Cdc24.^39^ Because these small GTPases are essential in many cellular events, for example, intracellular signal transduction, proliferation, inflammation and motility, great efforts have been made to identify compounds that inhibit prenylation with a view to their therapeutic use. The first suggestions that inhibitors of farnesylation could be used as anti-cancer drugs were made in 1982, with the identification of the RAS multigenic family as human oncogenes, and the subsequent demonstration that they required farnesylation to express their malignant transforming activity.^40^ Experimental studies confirmed the therapeutic potential of FTIs as, depending on the context, they induce apoptosis or cell cycle arrest, or inhibit cell proliferation, cell migration and angiogenesis.^41^ Consequently, at least 75 clinical trials have been conducted in various cancer indications since 2000, using four different FTIs, namely tipifarnib, lonafarnib, BMS-214662 and L-778123.^42^ However, most of these clinical trials have not been successful. For example, no significant survival gains were observed in patients with advanced solid cancers^43^ or acute myeloid leukemia.^44^ As most of these drugs have a very narrow therapeutic index, with toxicity developing at doses close to the therapeutic dose, one explanation for this failure could be the use of suboptimal doses in patients. The fact that Mono-APs were as effective as tipifarnib at inhibiting farnesylation at doses that did not induce cell toxicity, is encouraging and suggests that they may have a more favorable risk–benefit balance. However, this difference could also suggest that Mono-APs would be less effective antineoplastics than FTIs. Further experiments are required to compare Mono-APs with other FTIs in order to compare their potential interests against cancer cells. Another cause for failure may have been restriction of their activity to FT, as N-Ras and K-Ras may be prenylated through the geranylgeranylation alternative pathways in the presence of FTIs. This has led to the development of broad prenylation inhibitors for other diseases involving prenylated proteins, reported to target FPPS or HMGCR. To date, the FPPS inhibitors most commonly used in clinical practice are chemically stable analogs of inorganic pyrophosphate, all members of the bisphosphonate class.^45^ These compounds are mainly used to treat osteoporosis because they inhibit bone resorption. There are two major groups of bisphosphonates, with distinct molecular mechanisms. The first one comprises the non-nitrogen bisphosphonates, such as clodronate and etidronate, which mimic pyrophosphate and inhibit osteoclasts, ultimately causing their death, probably by interference with mitochondrial ATP translocases.^46^ The second group, the nitrogen-containing compounds such as zoledronate, interferes with specific metabolic reactions by inhibiting FPPS. They regulate a variety of cell processes controlled by these targets that are important for osteoclast function, including cell morphology, cytoskeletal arrangement, membrane ruffling, trafficking of vesicles and apoptosis.^47, 48^ More recently, a new type of FPPS inhibitor has been described, N6-isopentenyladenosine, which improves nuclear shape abnormalities in progeroid fibroblasts.^49^ In this study, we demonstrate that Mono-APs bind to both FPPS and FT, adding yet another mechanism of action, and targeting protein farnesylation at multiple sites. Any therapeutic use of Mono-APs will therefore need to be monitored carefully for adverse effects due to their lack of specificity, as with the existing prenylation inhibitors.

Since the discovery of human pluripotent stem cells, disease-specific cells have become a major tool for drug testing and discovery. Their well-acknowledged advantages over other cell models include their human origin, their non-transformed nature, their capacity for unlimited self-renewal and the theoretical possibility of deriving from them any cell phenotype of the body. When applied to drug discovery using HTS, pluripotent stem cell derivatives also offer the possibility of assaying phenotypic traits, instead of or in addition to specific biochemical readouts. Over the past 10 years, several studies have used this approach productively. Early drug screening on pluripotent stem cells focused on identifying compounds that would regulate stem cell maintenance^50, 51^ or cellular differentiation.^52, 53^ More recently, various authors have reported using phenotypic screening to identify drug candidates for specific diseases, such as cardiac hypertrophy^54^ or familial dysautonomia.^16^ In addition to all the advantages that already make them unique, it is worth highlighting the fact that these cells are amenable to functional testing. Secondary assays of the correction of pathological phenotypes, as described herein, may thus complement and provide physiological validation of an otherwise more reductionist primary drug screening assay. In the present study, it enabled us to immediately counterscreen all the candidate hit compounds for their ability to correct phenotypic traits associated with HGPS and to evaluate their toxicity in parallel. We could then select compounds that performed well in these tests for structure–activity relationship studies, in a cost and time effective manner.

## Materials and Methods

### Fibroblasts reprogramming

Fibroblasts used in this study were isolated from patient biopsies performed in the Assistance Publique Hôpitaux de Marseille for the patient 13-8243 and provided by Coriell Cell Repository (Camden, NJ, USA) for patients AG11513 and GM1972 and control DM4603 and AG8469. 13-8243 and DM4603 fibroblasts were reprogrammed to iPS cells using Yamanaka's original method with OCT4, KLF4, SOX2, c-Myc, transferred using retroviral vectors.^55^

### Pluripotent stem cell culture and differentiation

WT and HGPS iPS cells were grown on STO mouse fibroblasts, inactivated with 10 mg/ml mitomycin C seeded at 30 000/cm^2^ and grown as previously described.^19^ For differentiation, iPS cells were differentiated into MSCs using directed protocols for differentiation previously published by our group.^19^

### Prelamin A localization cell-based assay

2000 HGPS MSCs were plated in black 384-well clear-bottom plates (Corning, NY, USA). After 48 h of drug treatment, cells were fixed in 4% paraformaldehyde (15 min, room temperature). Permeabilization, blocking and primary hybridization steps were done concomitantly in a phosphate-buffered saline (PBS) solution supplemented with 0.1% triton X-100 and 1% bovine serum albumin (BSA; Sigma-Aldrich, St. Louis, MO, USA) and rabbit anti-prelamin A (ANTOO45, Diatheva, Fano, PU, Italy) overnight at 4 °C. Cells were stained with the species-specific fluorophore-conjugated secondary anti-rabbit antibody (Invitrogen, Carlsbad, CA, USA; 1 h, room temperature) and nuclei were visualized with Hoechst 33342. These steps were automated using a washing/staining station (ELx405 Biotek, Winooski, VT, USA, RapidStak Thermo Scientific, Waltham, MA, USA, Multidrop Labsystems by Thermo Scientific). Prelamin A localization was analyzed with an ArrayScan VTI HCS Reader (Cellomics Inc, Pittsburgh, PA, USA). The first channel was used for nucleus identification (Hoechst 33342 staining) and the second one to identify prelamin A staining. Pictures were acquired with a × 5 objective in high-resolution camera mode and were analyzed using the Spot Detector bioapplication. The robustness of the assay was evaluated using the Z′ factor calculated as follows Z′=1−[3(SDP+SDN)/(MP−MN)] where MP and MN correspond to the means of the positive (tipifarnib 1 *μ*M) and negative (DMSO 0.1%) controls, respectively, and SDP and SDN correspond to their S.D.

### Primary screening treatments

The primary screen was conducted on Biocell 1800 (Agilent, Santa Clara, CA, USA). For this, 2000 HGPS MSCs were seeded in 38 *μ*l of 20% FBS culture medium per well into black 384-well clear-bottom plates coated with 0.1% of gelatin. Five hours after seeding, 2 *μ*l of 20 × compounds from the chemical libraries were transferred in monoplicate into cell assay plates. In each plate, negative control (DMSO 0.1%) and positive control (tipifarnib 1 *μ*M) were added in columns 1 and 2, respectively. Plates were then incubated for 48 h and then processed for prelamin A detection assay. Each of the 72 plates of the screening were treated during 48 h, fixed and stained with the specific anti-prelamin A antibody using the optimized protocols as previously described. To prevent the discovery of toxic molecules, the number of cells per field was monitored in parallel and candidates showing mortality superior to 30% were excluded before their validation and their evaluation of phenotypical secondary phenotypes.

### Chemical library

Chemical library includes 21 608 compounds that belong to 4 different libraries distributed in 384-well plate format and obtained from Prestwick Chemical (Illkirch, France), LOPAC (Sigma-Aldrich), CHEM-X-INFINITY (Romainville, France) and Curie Institute (Orsay, France). The Prestwick Chemical library contains 1120 FDA approved drugs. This library was tested at two concentrations: 0.2 and 5 *μ*M. The LOPAC library (Sigma-Aldrich) contains a collection of 1280 pharmacologically active compounds. This library was tested at two concentrations: 0.2 and 10 *μ*M. The Chem-X-Infinity library is composed of 10 568 small organic molecules that belong to ~30 different chemical families. This library was tested at the concentration of 5 *μ*M. The Institute Curie library contains 8640 small molecules that were obtained during optimization programs against different therapeutic targets including anti-HIV, anti-kinases, protein–protein interactions, phosphatase inhibitors. This library was tested at 2.5 *μ*g/ml that corresponds to concentrations comprised between 2 and 15 *μ*M.

### Data analysis

Data analysis of the screening was performed using a customized Hiscreen application (Discngine, Paris, France) connected to Spotfire software (Tibco Software Inc., Palo Alto, CA, USA). Screening robustness was evaluated by calculating for each plate the Z′ factor on the percentage of prelamin A nuclei parameter. Raw data related to the percentage of cells with prelamin A nuclei and to cell number per field were normalized to the average of DMSO controls. Hits selection was performed using in parallel a *Z*-score plate and a *Z*-score run method on these normalized data. Only hits whose *Z*-score plate and/or *Z*-score run was ⩾3 and that did not decrease cell number by more than 30% compared with DMSO condition were selected for subsequent validation steps. These latter were retested in quadruplicate at the same concentration as for the primary screen. Validated hits were then tested at gradual concentrations for parallel exploration of their efficacy, potency and toxicity.

### Osteogenic differentiation

MSC were seeded at 600 cells per well in 384-well plate in MSC culture medium and treated as previously described. After 72 h, MSC medium was replaced by STEMPRO osteogenic induction medium (Invitrogen) in the presence or not of the different drugs. After 7 days of treatment, cells were fixed with ethanol 95% and stained by adding either a colorimetric substrate of the alkaline phosphatase, the 5-Bromo-4-chloro-3-indolyl phosphate/nitro blue tetrazolium (Sigma-Aldrich) or a chromogenic substrate of this enzyme (absorbance at 405 nm), the *p*-nitrophenyl phosphate (Pierce Biotechnology, Rockford, IL, USA).

### Immunocytochemistry

In the four steps protocol, cells were fixed in 4% paraformaldehyde (15 min, room temperature) before permeabilization and blocking in PBS supplemented with 0.1% triton X-100 and 1% BSA (Sigma-Aldrich). Primary antibodies were incubated 1 h at room temperature in blocking buffer. Antibodies included mouse anti-lamins A/C (clone JOL2, Millipore, Billerica, MA, USA), rabbit anti-prelamin A (ANTOO45, Diatheva) and anti-Ki-67 (clone Ki-S5, MAB4190, Millipore). Cells were stained with the species-specific fluorophore-conjugated secondary antibody (Invitrogen; 1 h, room temperature) and nuclei were visualized with Hoechst 33342.

### Western immunoblotting

Whole-cell lysates of MSC were collected, separated by SDS–PAGE and transferred onto polyvinylidene difluoride membrane by liquid transfer method. Blots were blocked in 10% skim milk (Bio-Rad, Hercules, CA, USA) in tween 0.1% tris-buffered saline (TTBS) 1 × 1 h at room temperature. The primary antibodies used were a mouse anti-lamins A/C 1:200 (Millipore, JOL2), a rabbit anti-prelamin A 1/100 (ANTOO45, Diatheva) and a *β*-actin 1/200000 (Sigma-Aldrich). Membranes were incubated during the night at 4 °C. Antigen–antibody binding was detected using horseradish peroxidase-conjugated species-specific secondary antibodies (GE-Healthcare, Little Chalfont, UK) followed by enhanced chemiluminescence western blotting detection reagents (Perkin-Elmer, Waltham, MA, USA).

### Quantitative PCR

Total RNA was isolated using RNeasy Mini extraction kit (Qiagen, Courtaboeuf, France) according to the manufacturer's protocol. An on-column DNase I digestion was performed to avoid genomic DNA amplification. RNA level and quality were checked using the Nanodrop technology. A total of 500 ng of RNA was used for reverse transcription using the Superscript III reverse transcription kit (Invitrogen). Q-PCR analysis was performed using a ABI 7900 system (Applied Biosystem, Foster city, CA, USA) and TaqMan gene expression Master Mix (Roche, Indianapolis, IN, USA) following the manufacturer's instructions. Quantification of gene expression was based on the DeltaCt Method and normalized on 18 S expression (Assay HS_99999901_S1). PCR primers were previously described by S. Rodriguez and colleagues (Rodrigez S *et al.*, 2009). Primer sequences were lamin A (exons 11/12), 5′-TCTTCTGCCTCCAGTGTCACG-3′ and 5′-AGTTCTGGGGGCTCTGGGT-3′ lamin C (exons 9/10), 5′-CAACTCCACTGGGGAAGAAGTG-3′ and 5′-CGGCGGCTACCACTCAC-3′ and Progerin (exons 11/12), 5′-ACTGCAGCAGCTCGGGG-3′ and 5′-TCTGGGGGCTCTGGGC-3′. Taqman MGB probe sequences were lamin A (exon 11), 5′-ACTCGCAGCTACCG-3′ lamin C (exon 10), 5′-ATGCGCAAGCTGGTG-3′ and Progerin (exon 11), 5′-CGCTGAGTACAACCT-3′. Reporter and quencher dyes for the LMNA locus assays were 5′-6FAM and 3′-nonfluorescent quencher dye (NFQ; Applied Biosystems).

### Molecular docking

The structural analysis and docking studies are based on crystal structure available from the Protein Data Bank. Structure of chain C and D of HMGCR were extracted from a complex with Pyrrole-based inhibitor (PDB id: 2Q1L, resolution of 2.05 Å). hFPPS was modeled using crystal structure (PDB id: 1YV5, resolution of 2.00 Å), including its three magnesium atoms but excluding its co-crystallized ligand. FT crystal structure (PDB id: 3E37, resolution of 1.80 Å) was used, keeping its zinc cation but removing its inhibitor. For both of these structures, hydrogen atoms were added and center of mass of experimental co-crystallized ligands were used to define the center of binding site, where conformational search was performed. AutoDock 4.2.1 was used for all docking calculations, using default values for docking parameters except the number of genetic algorithm (GA) runs, which was increased from 10 to 200 to increase conformational search. The resulting independent GA runs were then processed using clustering analysis with a 2.0 Å cutoff.

### HMGCR colorimetric activity assay

HMGCR activity was measured using HMG-CoA Reductase Assay Kit (Sigma-Aldrich) according to the manufacturer's protocol. The assay is based on the spectrophotometric measurement of the decrease in absorbance at 340 nm, which represents the oxidation of NADPH by the catalytic subunit of HMGCR in the presence of the substrate HMG-CoA.

### Preparation of cell lysates for FPPS and FT activity assays

Cell pellet of HGPS MSCs were resuspended in 25 mM Tris-HCl pH 7.4, 1 mM DTT, 1 mM MgCl_2_, 1 mM EDTA, 1 mM PMSF and centrifuged at 10 000 × *g* for 10 min at 4 °C. Protein content was determined using Lowry's method. Aliquots of cell lysate containing 100 *μ*g of total proteins were incubated with tested substances at 37 °C for 30 min.

### FPPS activity assay

FPPS assay was carried out with some modifications of the procedure of Krisans *et al.*^56^ and described by Gupta *et al.*^57^ Briefly, FPPS was assayed in 150 *μ*l containing 25 mmol/l Hepes, pH=7, 2 mmol/l MgCl2, 1 mmol/l dithiothreitol, 5 mmol/l KF, 1% noctyl-*β*-glucopyranoside, 3.3 *μ*mol/l [4-14C] IPP (18 Ci/mmol), 3 *μ*mol/l unlabeled IPP and 20 *μ*mol/l geranyl diphosphate. Reactions were started by adding 40 *μ*l of treated or untreated cell lysate and incubated for 45 min at 37 °C. Reactions were stopped by the additkion of 150 *μ*l 2.5 mol/l HCl in 80% ethanol containing 100 *μ*g/ml farnesol as a carrier. The samples were hydrolyzed for 30 min at 37 °C to convert the FPP to farnesol and neutralized by the addition of 150 *μ*l of 10% NaOH. The reaction product (farnesol) was extracted into 1 ml of N-hexane and an aliquot (200 *μ*l) of the organic phase was used for radioactivity counting. Parallel samples were assayed to evaluate the total and the nonspecific radioactivity. In all experiments, enzyme assays were carried out in triplicate.

### FT activity assay

FT activity was determined by FT [3H] SPA enzyme assay (Amersham Life Science, Buckinghamshire, England). Briefly, FT activity was assayed in 20 *μ*l of assay buffer containing 50 mmol/L Hepes, 30 mmol/l MgCl_2_, 20 mmol/l KCl and 5 mmol/l dithiothreitol, 20 *μ*l of [3H]-FPP (12 pmol) and 20 *μ*l of biotin lamin B. Reactions were started by adding 40 *μ*l of treated or untreated cell lysate and incubated for 1 hour at 37 °C. Reactions were stopped by the addition of 150 *μ*l of stop reagent containing SPA bead and counted in a scintillation counter. FT activity was expressed as pmoles of incorporated [3H]-FPP into biotin lamin B peptide per minute per milligram of total proteins (pmol/min/mg prot). Parallel samples were assayed to evaluate the total and the nonspecific radioactivity. In all experiments enzyme assays were carried out in triplicate.

### Surface plasmon resonance

SPR was measured using a BIAcore 3000 instrument at 25 °C. FPPS (2800 RU) was immobilized on flow cells of a CM5 chip. The running buffer was HBSEP pH 7.4 (20 mM Hepes, 150 mM NaCl, and 0.005% P20). Regeneration was performed with 10 mM glycine, pH 3.0. Measurements for affinity determinations of FPPS binders were performed with compounds injected at concentrations ranging from 1.95 nM to 5 *μ*M. Data were evaluated using Scrubber2 (Biologic Software, Campbell, ACT, Australia) and BIAeval Software (BIAcore by GE Healthcare, Little Chalfont, UK).

### Measure of Ras farnesylation

Ras farnesylation was measured by analyzing GFP localization in HGPS MSCs overexpressing a plasmid encoding the CaaX box of mammalian hRas fused to GFP at the C terminus.

### Live Cell Imaging

HGPS MSCs were seeded at 2500 cells per well in 96-well plates and cultivated during 7 days in presence of the drugs. Cell density was monitored using an IncuCyte ZOOM microscope (Essen Bioscience, Ann Arbor, MI, USA). Each value corresponds to the mean of cell densities of four pictures taken in three independent wells.

### Statistical analysis

Statistical analysis has been performed by one-way analysis of variance, using the Dunnet's comparison test. Values of *P*<0.05 were considered significant (*P<0.05, **P<0.01, ***P<0.001).

## Abbreviations

HGPS: Hutchinson-Gilford progeria syndrome
AP: aminopyrimidines
DNA: deoxyribonucleic acid
HMGCR: 3-hydroxy-3-methylglutaryl-CoA reductase
FPPS: farnesyl pyrophosphate synthase
FTI: farnesyl transferase inhibitor
HTS: high-throughput screening
ES: embryonic stem (cells)
iPS: induced pluripotent stem (cells)
VSMCs: vascular smooth muscle cells
MSCs: mesenchymal stem cells
DMSO: dimethyl sulfoxide
EC50: effective concentration
QC: quinoline carboxamide
SIMVA: simvastatin
IS: indole surrogate
SCM: spirochromane
GFP: green fluorescent protein
PCR: polymerase chain reaction
mRNA: messenger ribonucleic acid
WT: wild type
FT: farnesyl transferase
SPR: surface plasmon resonance
IC50: inhibitory concentration
TKI: tyrosine-kinase inhibitors
PBS: phosphate-buffered saline
BSA: bovine serum albumin
HCS: high-content screening
FDA: food and drug administration
TTBS: tween 0.1% tris-buffered saline
Q-PCR: quantitative PCR
PDB: protein data bank
GA: genetic algorithm
NADPH: nicotinamide adenine dinucleotide phosphate
DTT: dithiothreitol
EDTA: ethylenediaminetetraacetic acid
PMSF: phenylmethanesulfonylfluoride
IPP: isopentenyl pyrophosphate
