# Supplementary figures and images for: Drug screening on Hutchinson Gilford progeria pluripotent stem cells reveals aminopyrimidines as new modulators of farnesylation
Source: Cell Death Dis. 2016 Feb 18;7(2):e2105–. doi: 10.1038/cddis.2015.374 (PMC5399184; doi:10.1038/cddis.2015.374)

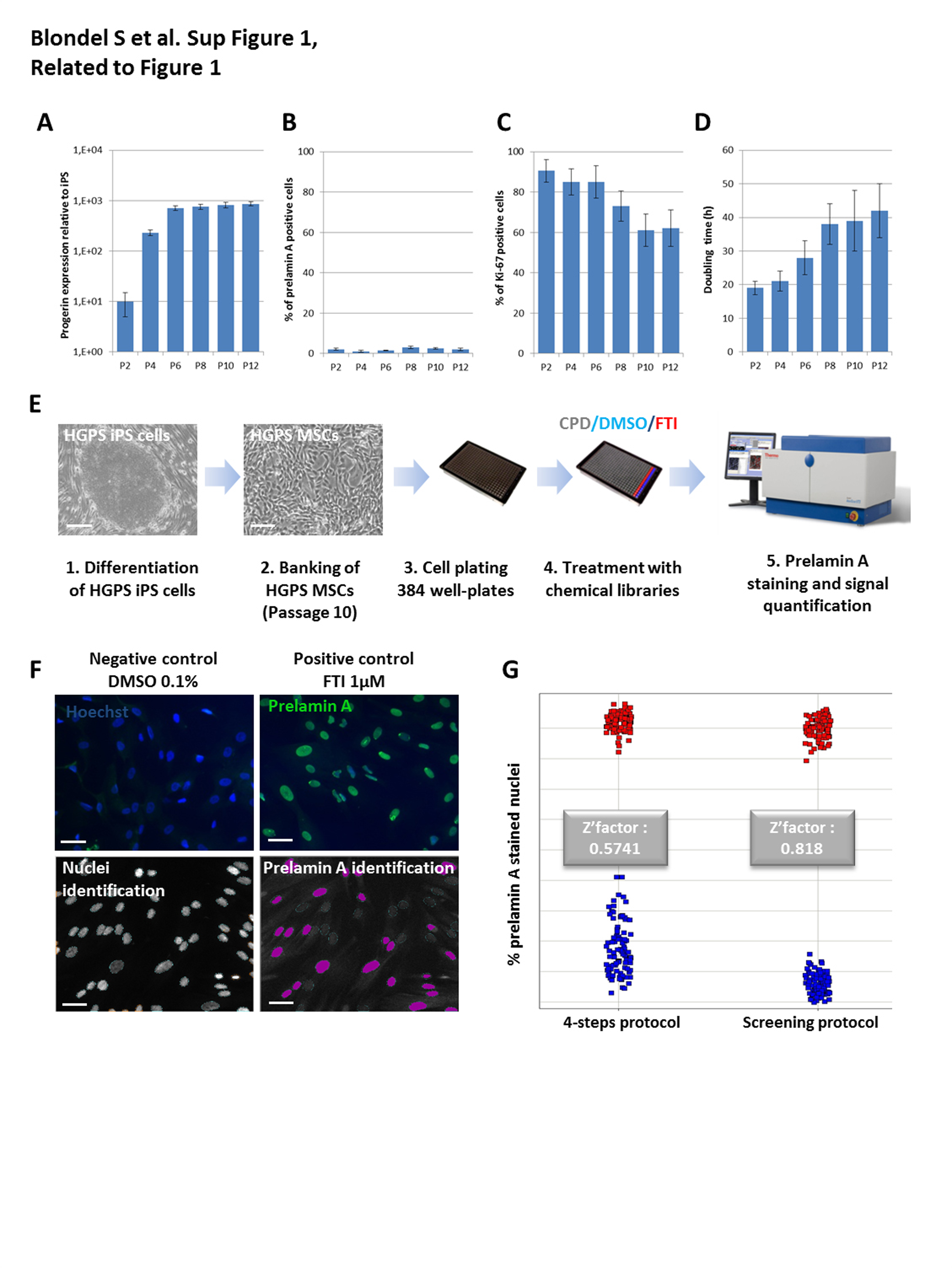

Supplement: Supplementary Figure 1 [file cddis2015374x1.tif]

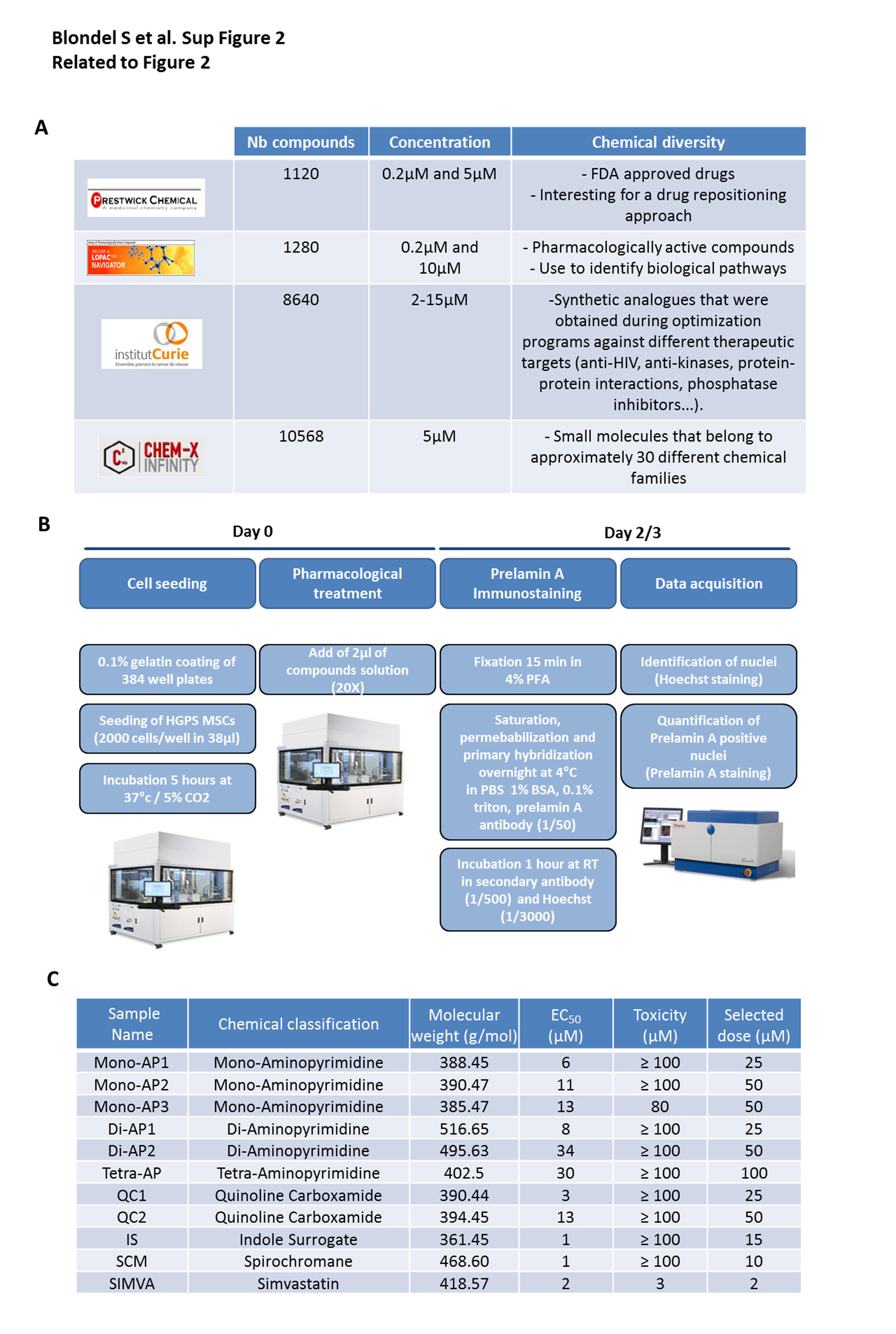

Supplement: Supplementary Figure 2 [file cddis2015374x2.tif]

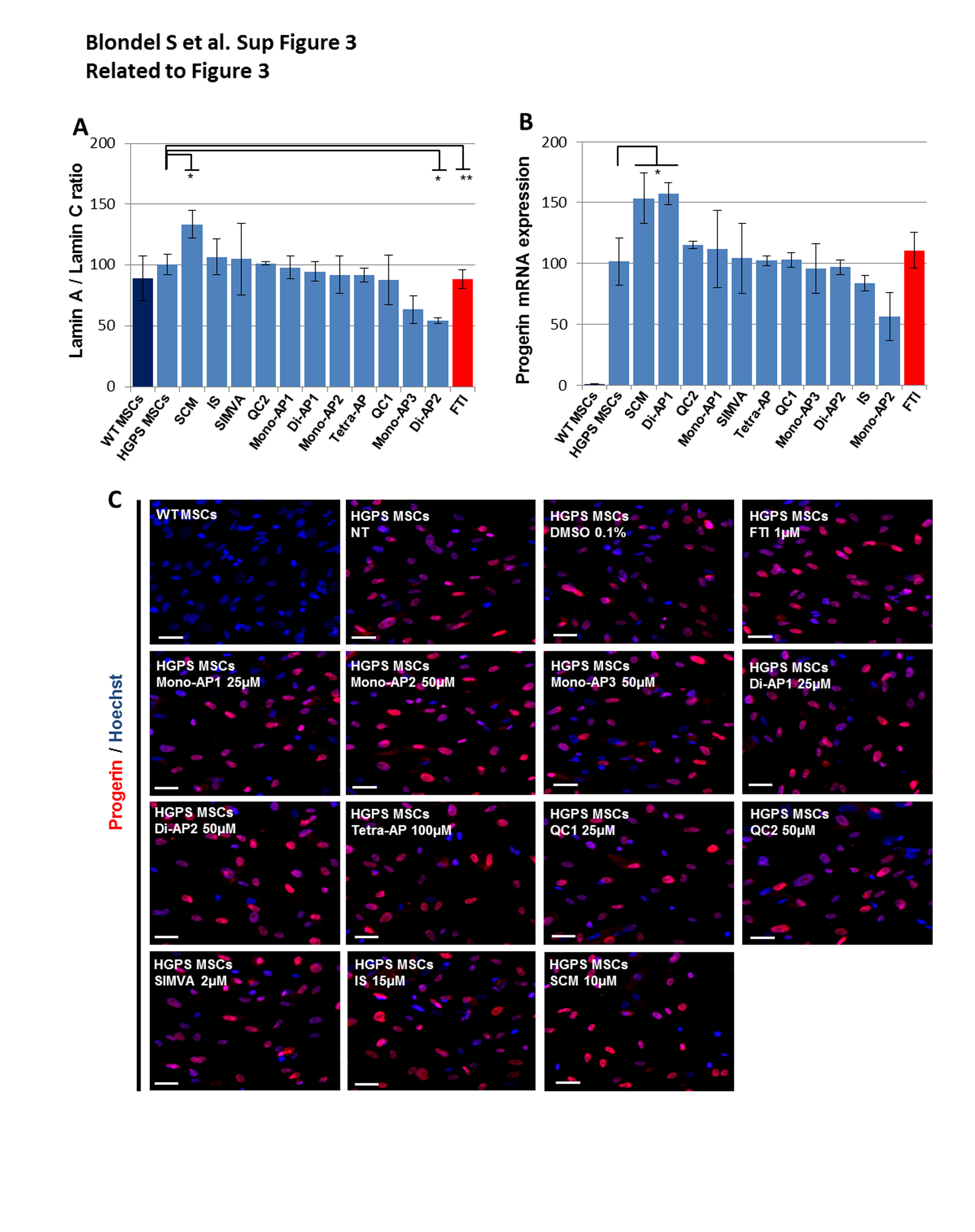

Supplement: Supplementary Figure 3 [file cddis2015374x3.tif]

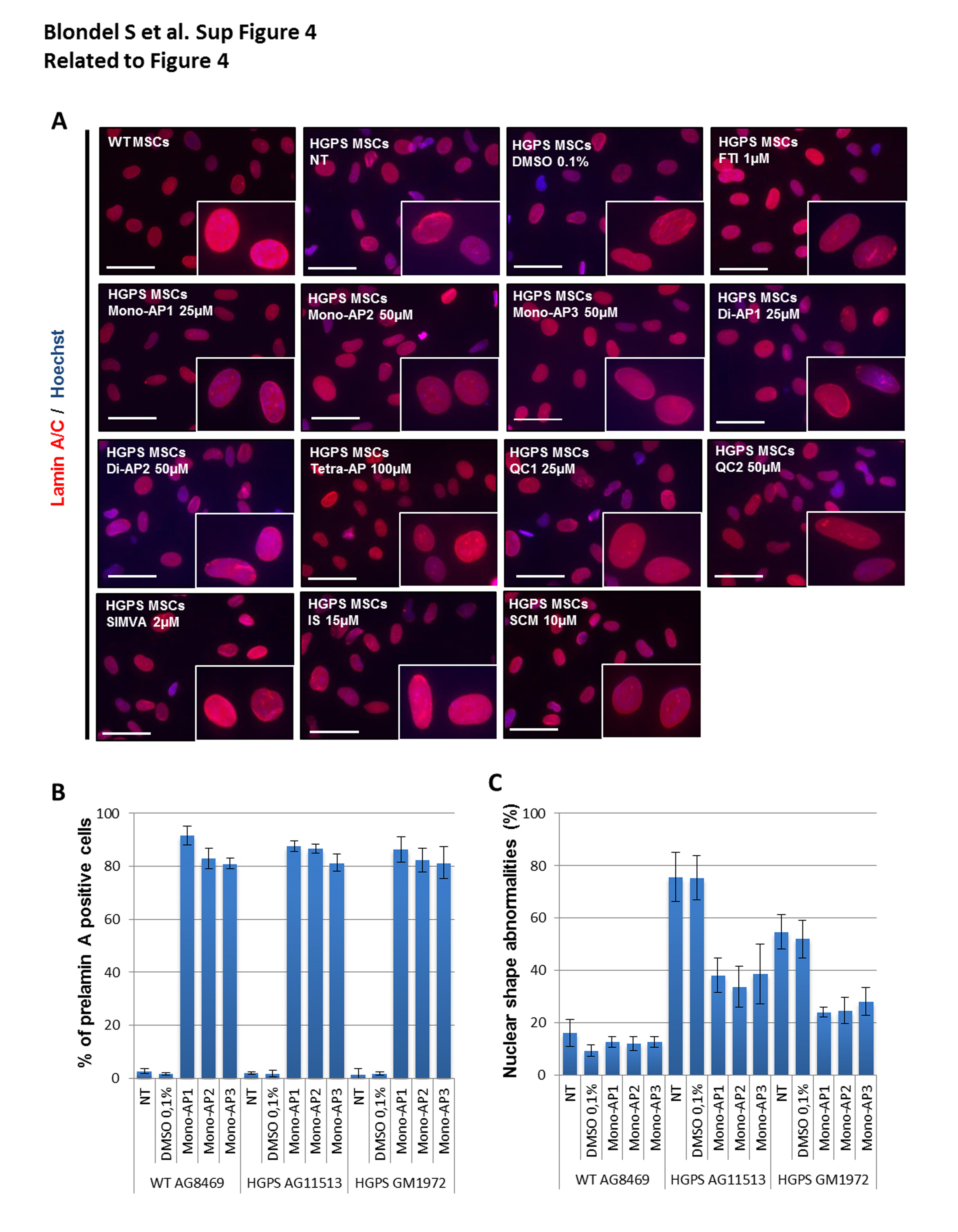

Supplement: Supplementary Figure 4 [file cddis2015374x4.tif]

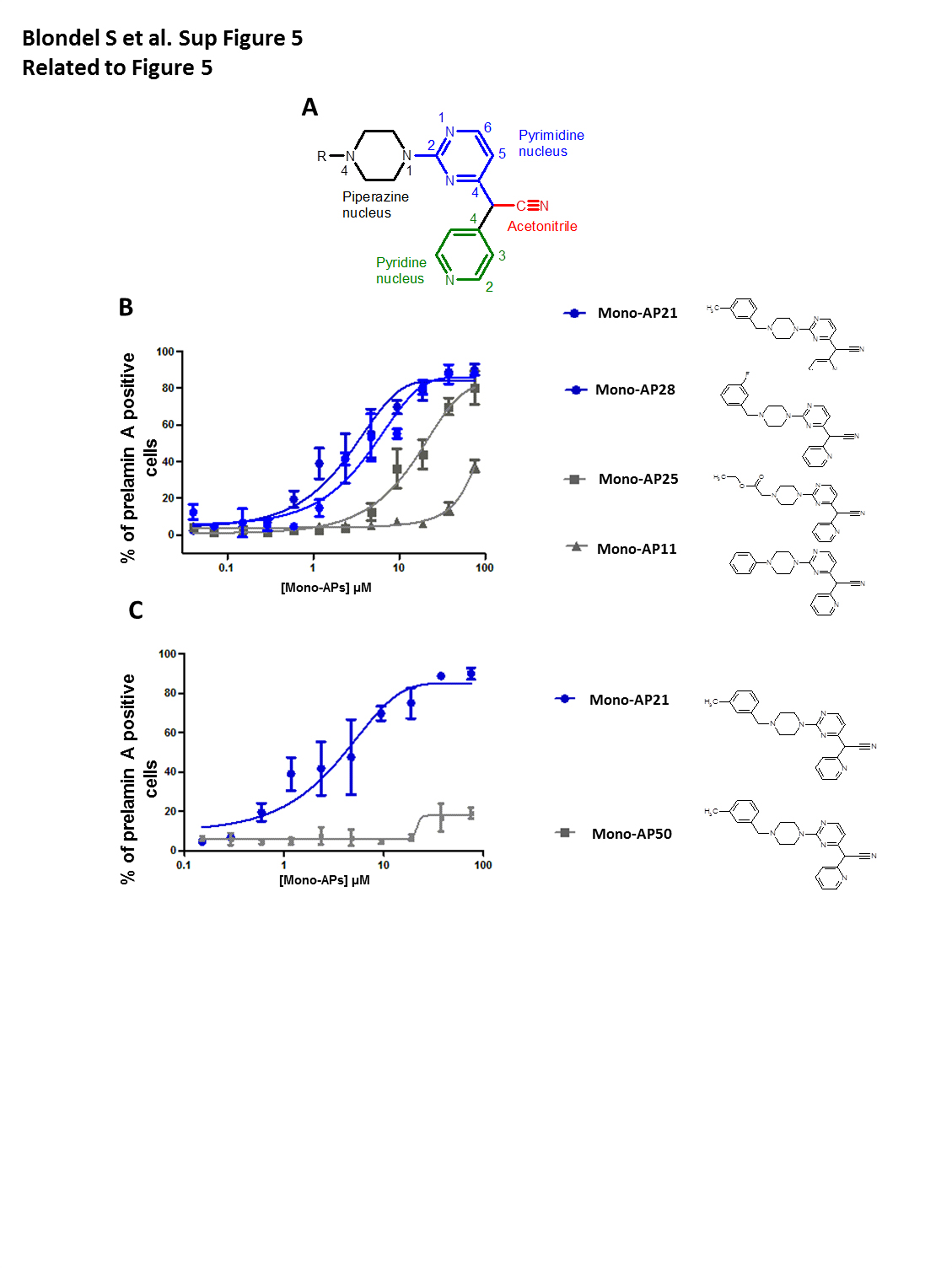

Supplement: Supplementary Figure 5 [file cddis2015374x5.tif]

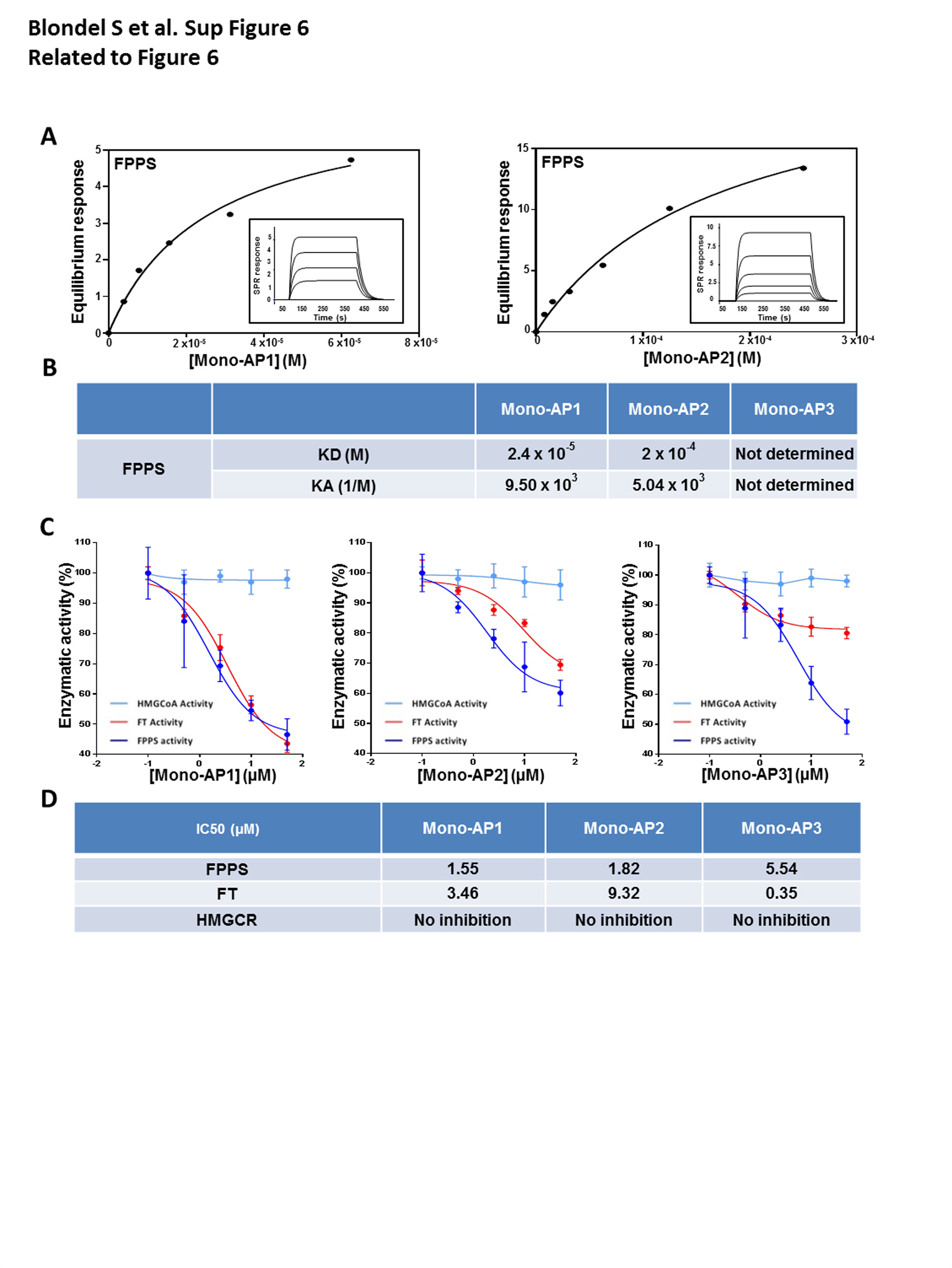

Supplement: Supplementary Figure 6 [file cddis2015374x6.tif]

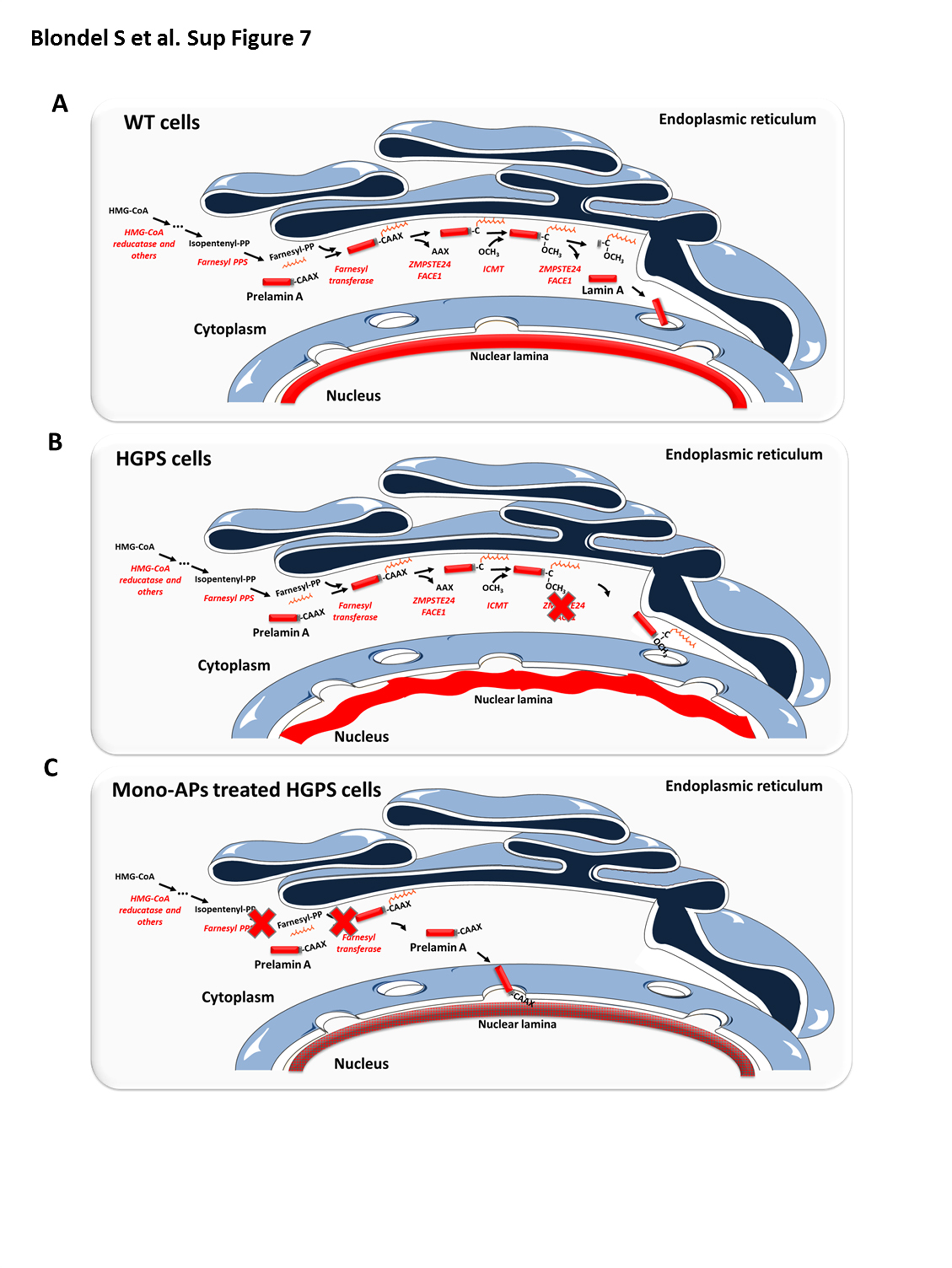

Supplement: Supplementary Figure 7 [file cddis2015374x7.tif]

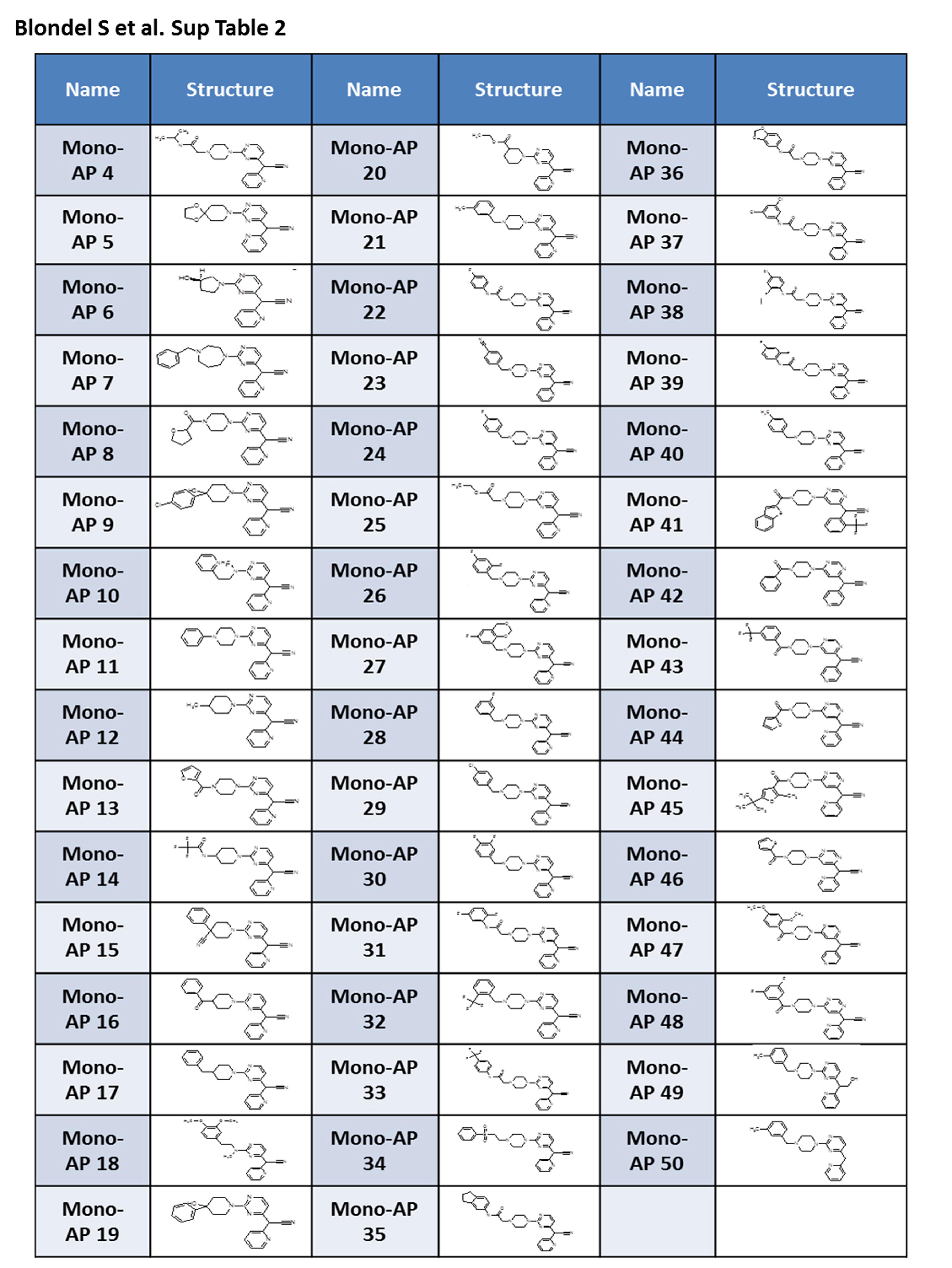

Supplement: Supplementary Table 2 [file cddis2015374x9.tif]
