# Supplementary material for: Drug screening on Hutchinson Gilford progeria pluripotent stem cells reveals aminopyrimidines as new modulators of farnesylation
Source: Cell Death Dis. 2016 Feb 18;7(2):e2105–. doi: 10.1038/cddis.2015.374 (PMC5399184; doi:10.1038/cddis.2015.374)
Supplement: Supplementary Table 1 [file cddis2015374x8.pdf]

Blondel et al Sup Table 1

SAMPLE_NAME	SAMPLE_ALIAS	% prelamin A positive nuclei	Toxicity	Comments
C280028-A	OIPZ1107209	92,6	-22,5	SCM
C245161-A	OIPZ1113672	90,8	-15,9	IS
C245805-A	OIPZ1140534	77,3	-13,8	Di-AP1
C245827-A	OIPZ1140005	64,3	-11,1	Di-AP2
C283051-A	P1300371	62,5	11,1	QC2
C249098-A	OIPZ1206803	55,7	-20,9	False positive
C285357-A	ICC109-L-004-E02-	54,2	-16,4	Weak effect
C286653-A	ICC121-L-020-G02-	52,5	-3,2	False positive
C285129-A	ICC115-L-001-F02-	50,6	-15,9	False positive
C280316-A	OIPZ1129985	47,8	-18,6	Mono-AP3
C247138-A	OIPZ1144244	47,7	1,5	Mono-AP1
C283057-A	P1300051	41,7	0,0	QC1
C000098-B	Prestw-98	40,7	-11,2	False positive
C282281-A	P1304433	38,9	-19,8	False positive
C248364-A	OIPZ1216843	37,7	-6,9	Weak effect
C249178-A	OIPZ1206608	37,6	-23,6	False positive
C248324-A	OIPZ1217316	37,6	-3,9	False positive
C000246-B	Prestw-246	35,1	-15,0	False positive
C281293-A	P1300211	34,3	-24,4	False positive
C246971-A	OIPZ1206136	34,3	-22,0	False positive
C281361-A	OIPZ1216844	34,1	-16,1	Toxic at the efficient concentration
C000179-B	Prestw-179	33,1	-15,1	False positive
C000019-B	Prestw-19	33	-19,7	False positive
C000257-B	Prestw-257	32,2	-0,5	False positive
C000859-B	Prestw-859	31,9	-14,7	False positive
C280274-A	OIPZ1128917	30,8	-25,3	False positive
C280034-A	OIPZ1107354	30,8	-23,6	False positive
C000244-B	Prestw-244	30,5	-16,0	False positive
C248859-A	OIPZ1254447	30,2	-15,1	Mono-AP2
C291907-A	ICC113-L-139-F10-	29,7	-3,7	Tetra-AP
C248920-A	OIPZ1216298	28,8	-13,4	False positive
C280079-A	OIPZ1107239	28,5	-7,3	False positive
C246442-A	OIPZ1204775	28,4	-20,3	False positive
C248321-A	OIPZ1217317	27,2	-29,3	False positive
C248966-A	OIPZ1214437	27,1	-20,7	False positive
C281046-A	OIPZ1244954	26,9	-19,0	False positive
C280022-A	OIPZ1107349	26,8	-29,8	False positive
C280242-A	OIPZ1124482	26,8	-24,0	False positive
C280026-A	OIPZ1107358	26,8	-19,4	False positive
C281030-A	OIPZ1243883	26,6	-16,5	False positive
C248963-A	OIPZ1216398	26,5	-11,2	False positive
C279491-A	OIPZ1119213	26,4	-28,8	False positive
C280276-A	OIPZ1129031	26	-25,4	False positive
C281016-A	OIPZ1243988	25,9	-16,5	False positive
C248325-A	OIPZ1217315	25,8	-21,0	False positive
C280261-A	OIPZ1124346	25,3	-21,6	False positive
C280263-A	OIPZ1124437	25,2	-20,1	False positive
C248965-A	OIPZ1214422	25	-16,0	False positive
C248947-A	OIPZ1216501	25	-11,0	False positive
C248336-A	OIPZ1217291	24,1	-8,9	False positive
C000865-B	Prestw-865	24,1	-20,0	SIMVA
C248968-A	OIPZ1214419	24	-24,5	False positive
C248384-A	OIPZ1219493	23,7	-11,5	False positive
C248969-A	OIPZ1214434	23,4	-12,1	False positive
C282519-A	P1304054	22,5	-19,1	False positive
C248090-A	OIPZ1235955	22,4	-17,1	False positive
C247253-A	OIPZ1224777	21,2	-20,8	False positive
C246441-A	OIPZ1204751	20,8	-16,9	False positive
C245744-A	OIPZ1129381	19,4	-17,9	False positive

(cyan)	Primary hit eliminated after dose response analysis
(pink)	Hits
