# Supplementary material for: Drug screening on Hutchinson Gilford progeria pluripotent stem cells reveals aminopyrimidines as new modulators of farnesylation
Source: Cell Death Dis. 2016 Feb 18;7(2):e2105–. doi: 10.1038/cddis.2015.374 (PMC5399184; doi:10.1038/cddis.2015.374)
Supplement: Supplementary Information [file cddis2015374x14.docx]

***Sup Figure 1: Development of a high throughput screening assay to quantify the effect of chemical compounds on prelamin A maturation in 384 well plates.***

1. Progerin expression in HGPS MSCs after two to twelve passages in culture.
2. Percentage of prelamin A positive cells in HGPS MSCs after two to twelve passages in culture.
3. Percentage of Ki-67 positive cells in HGPS MSCs after two to twelve passages in culture.
4. Doubling time of HGPS MSCs after two to twelve passages in culture.
5. Workflow of the high throughput screening of prelamin A modulators. Scale bar is 50µm.
6. Automated quantification of prelamin A-stained nuclei in HGPS MSCs following 48 hours of treatment with a FTI positive control (tipifarnib 1µM). Scale bar represents 50µm.
7. Determination of the Z’ factor following a 4-Steps immunostaining protocol and the screening protocol in 384 well plates. Each dot represents in blue negative controls (DMSO 0.1 %) and in red positive controls (tipifarnib).

***Sup Figure 2: Experimental workflow of the screening procedure identifying 11 inhibitors of prelamin A maturation***

1. Composition of the different chemical compounds libraries used in the screening.
2. Main steps of the screening procedure.
3. List of the 11 prelamin A modulators identified after screening validation. EC50 value corresponds to the dose inducing a 50 % increase of prelamin A positive cells. Toxicity corresponds to the dose inducing a 40 % decrease of the number of cells. Selected dose correspond to the dose used for the molecular characterization and secondary assays.

***Sup Figure 3: Experimental workflow of the screening procedure identifying 11 inhibitors of prelamin A maturation***

1. Gene expression analysis of lamin A/C ratio in HGPS MSC following 48 hours of treatments with each of the 11 validated compounds. Data are normalized on DMSO 0.1 % treated HGPS MSC. Each chart represents the mean +/- SD of three independent experiments.
2. Gene expression analysis of progerin expression in HGPS MSC following 48 hours of treatments with each of the 11 validated compounds. Data are normalized on DMSO 0.1 % treated HGPS MSC. Each chart represents the mean +/- SD of three independent experiments.
3. Immunostaining of progerin in HGPS MSCs following 48 hours of treatments with each of the 11 validated compounds. Scale bar represents 50µm.
4. Lamin A/C immunostaining of HGPS MSCs following 48 hours of treatments with each of the 11 validated compounds. Scale bar represents 50µm.

***Sup Figure 4: Effect of the 11 validated compounds nuclear shape abnormalities***

1. Typical lamin A/C immunostaining of nuclei considered as normal or not in HGPS MSCs.
2. Quantification of prelamin A maturation (prelamin A immunostaining) in WT (AG8469) and HGPS (AG11513, GM1972) fibroblasts following 48 hours of treatments Mono-AP1, Mono-AP2 and Mono-AP3. Each chart represents the mean +/- SD of 3 independent experiments.
3. Quantification of nuclear shape abnormalities (lamin A/C immunostaining) in WT (AG8469) and HGPS (AG11513, GM1972) fibroblasts following 48 hours of treatments Mono-AP1, Mono-AP2 and Mono-AP3. Each chart represents the mean +/- SD of 3 independent experiments.

***Sup Figure 5: Structure Activity Relationship study of Mono-APs***

1. Chemical structure of the different domains of Mono-APs.
2. Dose response analysis of Mono-AP21, Mono-AP28 in comparison to Mono-AP25 and Mono-AP11, on prelamin A maturation process in HGPS MSCs. Each point represents the mean +/- SD of the percentage of 8 replicates.
3. Dose response analysis of Mono-APs containing (Mono-AP21) or not (Mono-AP50) the nitrile group on prelamin A maturation process in HGPS MSCs. Each point represents the mean +/- SD of the percentage of 8 replicates.

***Sup Figure 6: Determination of pharmacological parameters of Mono-APs inhibitions of FPPS and FT***

1. Determination of the dissociation constant for Mono-AP1 and Mono-AP2 bindings to immobilized FPPS. Boxed charts represents raw SPR sensorgrams of Mono-AP1 and Mono-AP2 bindings to immobilized FPPS at compounds concentrations ranging from 1,5µM to 50µM. Equilibrium SPR intensity is plotted as a function of compounds concentrations.
2. Dissociation constants of Mono-AP1 and Mono-AP2 interactions with FPPS.
3. Dose response analysis of FPPS, FT and HMGCR activities in presence of Mono-AP1, Mono-AP2 and Mono-AP3. Results are presented in percent of control. Each point represents the mean +/- SD of the percentage of 3 replicates.
4. Values of IC50s of Mono-APs on FPPS, FT and HMGCR enzymatic activities.

***Sup Figure 7: Schematic representation of Mono-APs mode of action on prelamin A maturation process in HGPS cells***

1. Farnesyl PP is produced from HMG CoA through a cascade of enzymatic reaction including among others, two enzymes HMG CoA reductase and farnesyl pyrophosphate synthase. Farnesyl PP is the donor of the farnesyl group that permits to the prelamin A to be anchored to the endoplasmic reticulum membrane for its maturation. This transfer is performed by farrnesyl transferase. In WT cells, after successive steps of maturation, the farnesyl group is cleaved from prelamin A releasing mature lamin A into nuclear lamina.
2. In HGPS cells this final step of prelamin A maturation process (cleavage of the farnesyl group) is not possible because of the deletion of the recognizing site of the enzyme which cleave the C-terminal part of the farnesylated protein (ZMPSTE24/FACE1).
3. Our results revealed that Mono-APs are inhibiting both the production of the farnesyl PP synthesis through inhibition of farnesyl pyrophosphate synthase and also the transfer of the remaining farnesyl group to prelamin A through inhibition of farnesyl transferase. This dual inhibition avoid its anchoring in the endoplasmic reticulum and leads to the translocation of the immature protein into the nuclear lamina.

***Sup Table 1: List of the 59 molecules identified as hits during the screening***

***Sup Table 2: Chemical structure of the 47 Mono-APs analogous derived from Mono-AP.***

**Sup Video 1: 3D docking of Mono-AP3R on FPPS**

**Sup Video 2: 3D docking of Mono-AP3S on FPPS**

**Sup Video 3: 3D docking of Mono-AP3R on FT**

**Sup Video 4: 3D docking of Mono-AP3 on FT**
